# Purification of specific DNA species using the CRISPR system

**DOI:** 10.1093/biomethods/bpz008

**Published:** 2019-07-08

**Authors:** Toshitsugu Fujita, Hodaka Fujii

**Affiliations:** Department of Biochemistry and Genome Biology, Hirosaki University Graduate School of Medicine, Zaifu-cho 5, Hirosaki, Aomori, Japan

**Keywords:** CRISPR, enChIP, dCas9, NGS, DNA purification

## Abstract

In 2013, we developed a new method of engineered DNA-binding molecule-mediated chromatin immunoprecipitation that incorporates the clustered regularly interspaced short palindromic repeats (CRISPR) system to purify specific DNA species. This CRISPR-mediated purification can be performed in-cell or *in vitro*; CRISPR complexes can be expressed to tag target DNA sequences in the cells to be analyzed, or a CRISPR ribonucleoprotein complex consisting of recombinant nuclease-dead Cas9 (dCas9) and synthetic guide RNA can be used to tag target DNA sequences *in vitro.* Both methods enable purification of specific DNA sequences in chromatin structures for subsequent identification of molecules (proteins, RNAs, and other genomic regions) associated with the target sequences. The *in vitro* method also enables enrichment of purified DNA sequences from a pool of heterogeneous sequences for next-generation sequencing or other applications. In this review, we outline the principle of CRISPR-mediated purification of specific DNA species and discuss recent advances in the technology.

## Introduction

The advent of clustered regularly interspaced short palindromic repeats (CRISPR) technology for genome editing in 2012 [1, 2] opened a wide range of potential applications [3], among them is the purification of specific genomic regions from cells for subsequent biochemical analysis [4]. Several methods had already been developed for this purpose, as discussed in our previous review [5], but all of these methods have drawbacks. For example, tagged oligonucleotide probes can be used to purify specific genomic regions [6], but this requires partial denaturing of chromatin, making it difficult to achieve high yields for analysis of a single copy gene. It is also possible to insert the recognition site of an exogenous DNA-binding molecule such as the bacterial protein LexA into a genomic region of interest and use it as a tag for affinity purification [7], but this requires the laborious and time-consuming step of generating cells harboring the tagged locus. In addition, insertion of exogenous DNA may disturb the physiological conditions around the insertion site.

To overcome these problems, we developed a general strategy to purify specific DNA species using engineered DNA-binding molecules such as zinc-finger proteins (ZFPs), transcription activator-like (TAL) proteins, and the CRISPR system [4], and called this technology engineered DNA-binding molecule-mediated chromatin immunoprecipitation (enChIP). CRISPR technology provides an ideal system for flexible tagging of DNA to be purified. For this purpose, mutant forms of Cas9 lacking nuclease activity but retaining DNA-binding activity (nuclease-dead Cas9, dCas9) are used. Because the specificity of binding to target DNA can be directed by guide RNAs (gRNAs), generation of sequence-specific binding molecules is easier, cheaper, and requires less time than protein-based systems using ZFPs and TALs for which target-specific DNA-binding proteins must be generated each time. These advantages make our CRISPR-based system the method of choice for various applications, including DNA purification.

In addition to purification of specific genomic regions from cells, CRISPR-mediated purification of specific DNA species also enables enrichment of purified DNA sequences from a large number of heterogeneous sequences [8] and may be useful for various applications including clinical diagnosis.

In this review, we outline the principle of CRISPR-mediated purification of specific DNA species and discuss recent advances in the technology.

## Principles of CRISPR-mediated purification of specific DNA species

The two methods for CRISPR-mediated purification of specific DNA species differ in whether target DNA is tagged in the cell or *in vitro*.

### In-cell tagging

**Figure 1:**
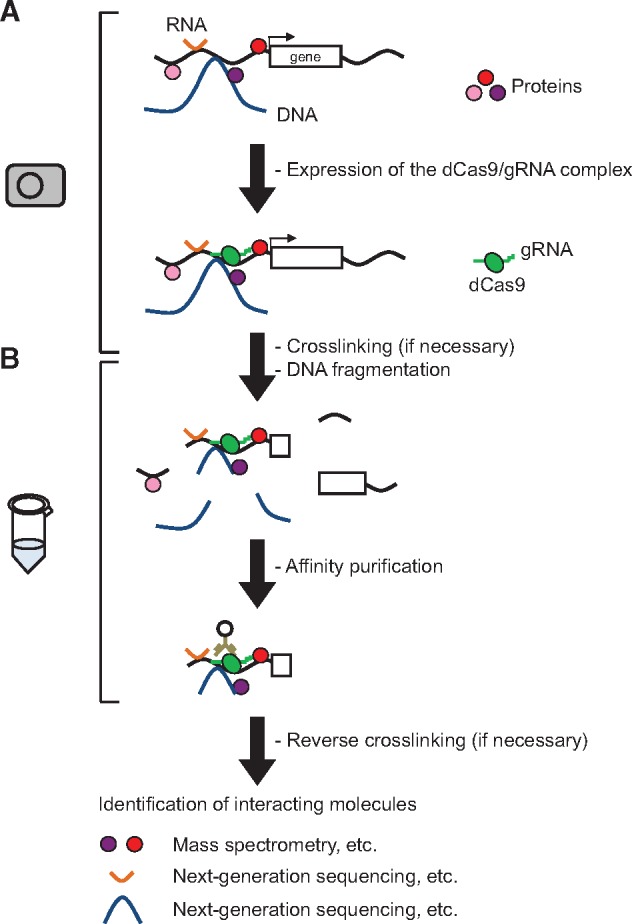
In-cell tagging of specific genomic regions using the CRISPR complex. (**A**) A locus of interest is tagged with CRISPR complex, consisting of a catalytically inactive form of Cas9 (dCas9) and gRNA that recognizes the target sequence, that is expressed in the cells to be analyzed. (**B**) Intermolecular interactions can be stabilized with a cross-linker such as formaldehyde, if necessary. Subsequently, chromatin is fragmented by sonication or enzymatic reactions. The target genomic region is isolated by affinity purification. After reverse cross-linking if necessary, molecules interacting with the target genomic region are identified by MS (proteins) and NGS (RNAs and other genomic regions).

In this method, target DNA is tagged in the cell, typically in the nucleus, by expression of the CRISPR complex (Fig. 1). Expression of the CRISPR complex can be achieved by (i) expression from DNA via transient or stable transfection of expression plasmids, establishment of stable transformants using retroviral or lentiviral vectors, or long-term continuous expression by adenovirus systems; (ii) expression from RNA after transfection of mRNA encoding dCas9 and gRNA (synthetic or *in vitro* transcribed); or (iii) transduction of CRISPR ribonucleoprotein (RNP) complexes consisting of recombinant dCas9 and synthetic gRNA. DNA-mediated expression, particularly sustained expression by retroviral, lentiviral, or adenoviral vectors, may be better for generating sufficient numbers of cells for analysis than RNA transfection or RNP transduction.

The in-cell approach permits targeting of cellular genomic DNA as well as parasitic DNA, which may include viral DNA such as hepatitis B virus and episomal DNA such as human papilloma virus. DNA reverse transcribed from an RNA virus and integrated into the host genome can also be targeted. Usually, these DNAs are associated with histones and other DNA-binding proteins as well as RNA and other genomic regions to form chromatin structures. After DNA purification, mass spectrometry (MS) can be used to identify proteins associated with the target DNA and next-generation sequencing (NGS) can be used to identify RNA and other genomic regions.

### *In vitro* tagging

**Figure 2:**
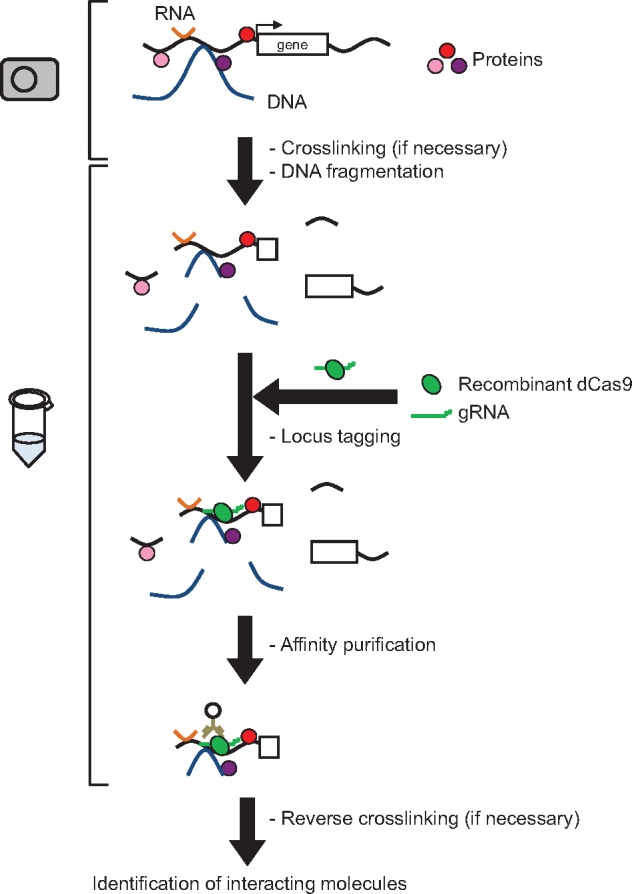
*In vitro* tagging of specific genomic regions using the CRISPR complex. Recombinant dCas9 protein is produced in an appropriate expression system such as *Escherichia coli* or silkworm, and gRNA is chemically synthesized, and then the two are mixed in a test tube to form functional CRISPR complexes. Subsequently, fragmented chromatin (native or fixed) is incubated with the CRISPR complexes for tagging. After affinity purification, molecules associated with the target DNA are identified by MS (proteins) or NGS (RNAs and other genomic regions).

In this method, target DNA is tagged *in vitro* using CRISPR complexes consisting of recombinant dCas9 and synthetic gRNA (Fig. 2). This method is applicable for targets in purified DNA and in chromatin DNA. One advantage of *in vitro* tagging is that expression of CRISPR complexes in target cells is not necessary, so less time and effort is required for DNA purification. This is especially important when purifying a target from primary cells such as clinical samples. *In vitro* tagging also avoids potentially hazardous effects caused by expression and binding of CRISPR complexes to genomic DNA, which may include inhibition of gene expression (CRISPR interference effect) and changes in chromatin accessibility [9]. One drawback of *in vitro* tagging, however, is that the yield of affinity purification is significantly lower than that for in-cell tagging [8]. It is possible that during *in vitro* tagging, only a fraction of target sites are accessible to CRISPR complexes, whereas in-cell tagging permits binding during the entire cell cycle. The feasibility of expressing CRISPR complexes and the number of obtainable cells should be considered when deciding which method of tagging to use.

**Figure 3:**
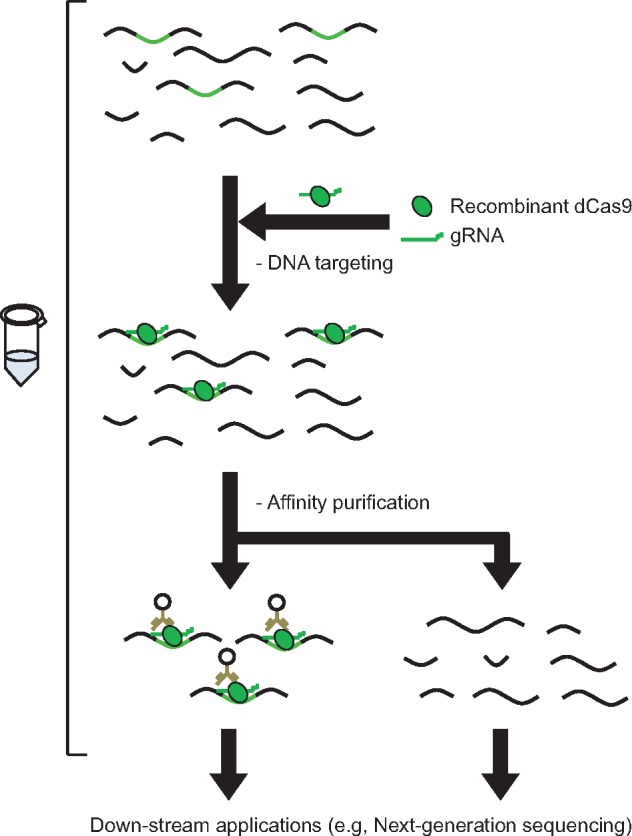
*In vitro* tagging of purified DNA using the CRISPR complex. DNA of interest in a pool of purified DNA species can be tagged using functional CRISPR complexes consisting of recombinant dCas9 protein and synthesized gRNA. Selected DNA species can then be analyzed by downstream applications such as NGS.

In addition to purification of DNA from chromatin, *in vitro* tagging enables isolation of specific DNA species from a heterogeneous mixture of purified DNA molecules (Fig. 3) [8]. Potential applications include target enrichment for NGS analysis, enrichment of pathogenic DNA from clinical specimens to enable more efficient diagnosis, and enrichment of exogenous DNA integrated during gene therapy and insertional mutagenesis to identify integration sites. *In vitro* tagging can also be used to remove undesirable DNA species, such as reverse-transcribed ribosomal RNA and mitochondrial DNA before transcriptome analysis and *16S rDNA* species from mitochondria, chloroplasts, or dominant bacteria during *16S rDNA*-based microbiome analysis, among others. Such use decreases the number of reads necessary to achieve sufficient quality of NGS analysis and reduces cost, although reports of such applications are lacking.

## Downstream applications of CRISPR-mediated DNA purification

**Figure 4:**
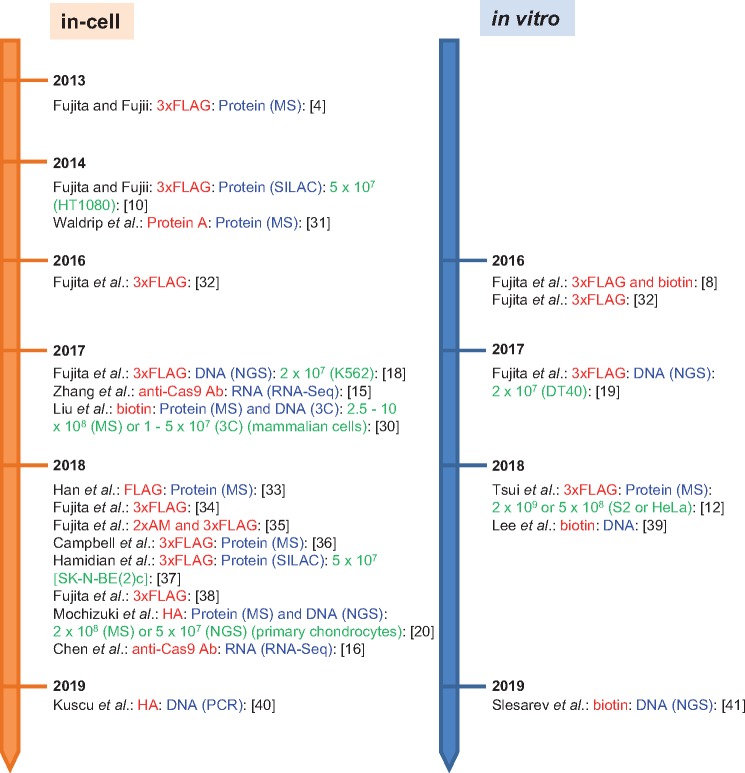
Publications reporting CRISPR-mediated purification of specific DNA species. Epitope tags used for affinity purification and target molecules (and downstream applications) are shown in red and blue [31–34, 36–38, 40, 41]. The CRISPR system of *Streptococcus pyogenes* (*Sp*) was used in all studies. In addition, the CRISPR system of *Staphylococcus aureus* (*Sa*) was used in one study [35]. An updated list of publications, including those using other engineered DNA-binding molecules, can be found at: http://www.med.hirosaki-u.ac.jp/~sim;bgb/iChIP_related_papers/enChIP/index_e.html.

Since our first report of CRISPR-mediated purification of specific DNA species in 2013 [4], an increasing number of applications of our strategy have been reported (Fig. 4). We discuss these applications below.

### Identification of proteins associated with a specific genomic region

CRISPR-mediated purification of specific DNA species can be combined with MS analysis to identify proteins associated with target DNA sequences. In our original report, we targeted the promoter of the *interferon regulatory factor-1(IRF-1)* gene [4, 10] and used a shotgun MS approach and stable isotope labeling using amino acids in cell culture (SILAC), a quantitative form of MS [11]. Many studies have used in-cell locus tagging with the CRISPR complex followed by MS to identify proteins associated with specific genomic regions (Fig. 4). *In vitro* tagging has also successfully been used for MS-mediated identification of proteins associated with a target genomic region [12].

### Identification of RNAs associated with a specific genomic region

We have shown that TAL protein-mediated purification of specific DNA species (enChIP using TAL proteins) combined with RT–PCR or RNA sequencing enables detection and identification of RNAs associated with specific genomic regions [13, 14]. CRISPR-mediated purification of specific genomic regions can also be combined with RNA sequencing to identify RNAs associated with target genomic regions [15, 16].

### Identification of intragenomic interactions with a specific genomic region

Investigation of regulation of nuclear organization has revealed that physical interactions between different genomic regions can play critical roles in genome functions such as transcription. Techniques to detect such physical interactions between genomic regions have been developed and reviewed previously [17]. We showed that CRISPR-mediated purification of specific DNA species combined with NGS, a process we call enChIP-Seq, allows identification of physical interactions between genomic regions [18, 19]. We used both in-cell [18] and *in vitro* tagging [19] with the CRISPR complex, and by combining enChIP-Seq, epigenetic profiling of the genomic regions interacting with a target gene promoter, and CRISPR-mediated locus deletion, we successfully identified a potential enhancer located in a different chromosome from the target gene promoter [19]. Subsequently, the same approach was used to identify a functional distant enhancer of the *Sox9* gene [20]. These results demonstrate that CRISPR-mediated locus purification combined with NGS is a powerful tool to identify physical interactions between genomic regions.

There are several different approaches to detect physical interactions between genomic regions. The development of chromosome conformation capture (3C) [21] spurred innovation of biochemical detection methods, and many derivatives of 3C have been reported [22]. 3C and related methods depend on ligation of DNA fragments in interacting chromatin complexes, but the ligation step can be a source of noise in the detection of interactions. Therefore, an increasing number of “ligation-free” biochemical methods have been developed, including enChIP-Seq, the in-gel replication of interacting DNA segments (INGRID) assay [23], the split-pool recognition of interactions by tag extension (SPRITE) approach [24], and chromatin interaction analysis via droplet-based and barcode-linked sequencing (ChIA-Drop) [25] (Table 1). INGRID, SPRITE, and ChIA-Drop all require isolation of a single chromatin complex consisting of different genomic regions. INGRID isolates the complex by its position in a layer of polyacrylamide gel, SPRITE uses a repeat of terminal barcoding of different genomic regions and shuffling, and ChIA-Drop depends on barcoding of different genomic regions in gel-bead-in-emulsion (GEM) droplets.

**Table 1:** Ligation-free biochemical methods to detect interactions between genomic regions

Method	Interactions (n-to-n)	Ref #
INGRID	One-to-one	[23]
enChIP-Seq	One-to-all	[18, 19]
SPRITE	All-to-all	[24]
ChIA-Drop	All-to-all	[25]

Of these methods, enChIP-Seq, SPRITE, and ChIA-Drop are non-biased approaches. INGRID is a “one-to-one” approach, whereas enChIP-Seq is a “one-to-all” approach, and SPRITE and ChIA-Drop are “all-to-all” approaches (Table 1). The enChIP-Seq procedure is much simpler than the procedures of SPRITE and ChIA-Drop; because enChIP-Seq using CRISPR is essentially ChIP-Seq of the CRISPR complex, it would not be difficult for anyone experienced with ChIP-Seq to perform. In addition, if the goal is to identify “one-to-all” chromosomal interactions such as identification of enhancers interacting with a target promoter region, enChIP-Seq would be more straightforward and cost effective than SPRITE and ChIA-Drop.

In addition to the biochemical methods discussed, imaging approaches such as fluorescence *in situ* hybridization [26] and live imaging of specific genomic loci [27–29] have also been reported. Although these imaging techniques do not provide non-biased detection of interactions between genomic regions, they are valuable for confirming results obtained by 3C-derived methods as well as other ligation-free biochemical methods.

## Tag systems, cell numbers, and other experimental conditions used for affinity purification

As shown in Fig. 4, many tag systems are compatible with CRISPR-mediated purification of DNA. Although one report claimed that a biotin tag led to a higher yield than a 1xFLAG tag and anti-Cas9 antibody [30], the authors did not compare the biotin tag with other high-affinity tags such as a 3xFLAG tag. We obtained very high yields using CRISPR-mediated purification of DNA with 3xFLAG-fused dCas9, suggesting that other high-affinity tag systems would also be suitable.

Cell numbers used for CRISPR-mediated purification of DNA vary between experiments. In general, however, identification of proteins associated with a specific genomic region requires more cells than identification of nucleic acids. In total, 5 × 10^7^ to 2 × 10^9^ cells were used for protein identification, whereas 1 × 10^7^ to 5 × 10^7^ cells were used for identification of associated genomic regions (Fig. 4).

Few studies have described the amounts of proteins and nucleic acids used for MS and NGS analyses, respectively. We used 1–5 ng of purified DNA for NGS analysis of genomic regions associated with a specific genomic region [19]. Other reports used 5 ng of DNA for 3 C analysis [30] and 10 ng of DNA for NGS [20].

## Concluding remarks

In this review, we summarized recent advancements in CRISPR-mediated purification of specific DNA sequences. When this technology is applied to cellular DNA, molecules binding to the target DNA can be identified by MS and NGS. When it is used with purified DNA, target DNA species can be enriched or removed for downstream analyses. This technology will be useful in chromatin and epigenetics research as well as targeted genomics and transcriptomics.
